# Supplementary material for: Functional Connectivity between the Resting-State Olfactory Network and the Hippocampus in Alzheimer’s Disease
Source: Brain Sci. 2019 Nov 25;9(12):338. doi: 10.3390/brainsci9120338 (PMC6955985; doi:10.3390/brainsci9120338)
Supplement: Supplementary file 1 [file brainsci-09-00338-s001.pdf]

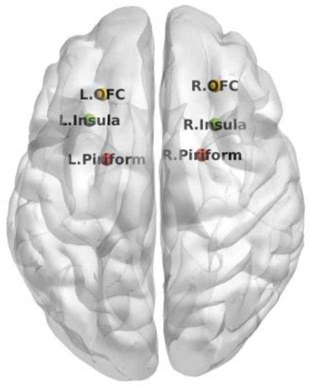

Figure S1. Olfactory network (ON) was defined based on published fMRI activation studies. Time courses were extracted in MNI space [(x y z) coordinates] from these six regions as the average time course within a five voxel radius centered on the following coordinates: POC ([−22 0 −14], [22 2 −12]), OFC ([−24 30 −10], [28 34 −12]), and insula ([−30 18 6], [28 16 8]).

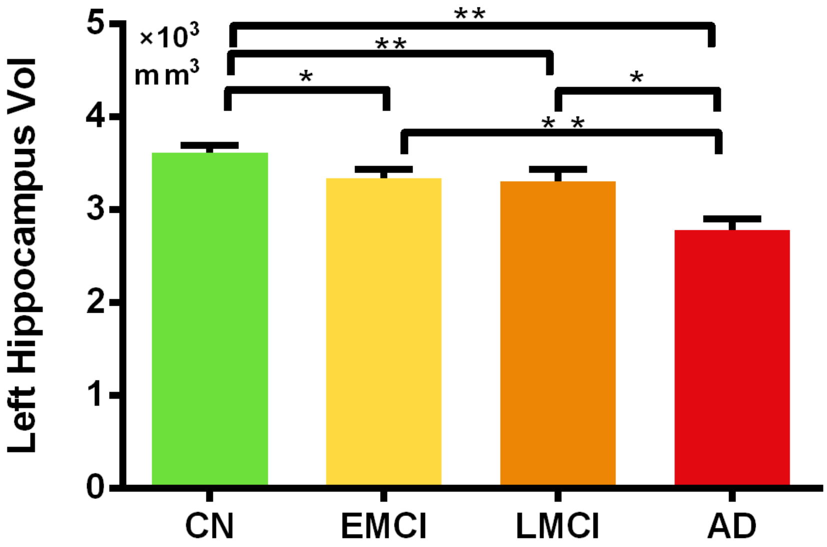

Figure S2. Left hippocampal volume in CN (cognitively normal), EMCI (early mild cognitive impairment), LMCI (late mild cognitive impairment), and AD (Alzheimer's disease) groups. *: $p<0.05$; **: $p<0.01$

Degree centrality analysis

The degree centrality (DC) is the number of direct connections of a given voxel. This measure has been widely used to represent node characteristic of large-scale, intrinsic brain networks. In this paper we used preprocessed resting state fMRI data to calculate the DC of hippocampal voxels. The time course of each voxel was correlated with every other voxel time course within a gray matter mask and thresholded at a correlation coefficient of $r > 0.25$ [1]. The DC at each hippocampal voxel was then calculated as the sum of significant correlation coefficient (weighted DC). Finally, these voxel-wise DC values were transformed into a Z-scores using the Fisher-Z transformation. Because of the uncertainty of interpretation and detrimental effects on test retest reliability, only the positive correlations were considered in DC calculation.

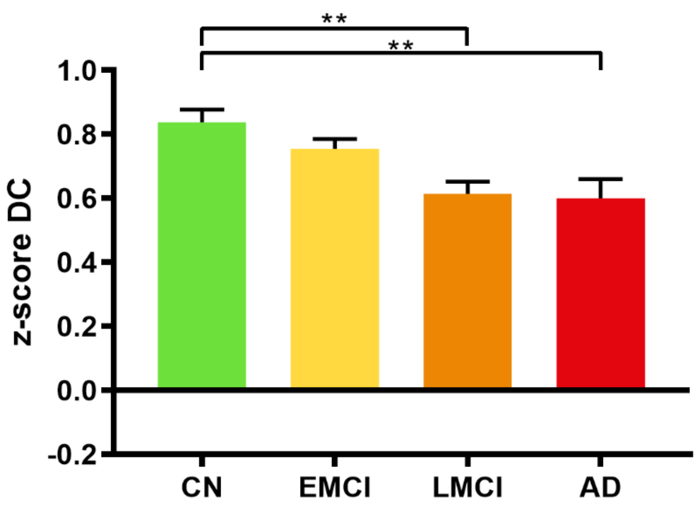

Figure S3. DC values in the right hippocampus in CN (cognitively normal), EMCI (early mild cognitive impairment), LMCI (late mild cognitive impairment), and AD (Alzheimer's disease) groups. DC values in the right hippocampus are decreasing dependent on AD disease state. This demonstrates potential isolation of the right hippocampus from the rest of the brain during AD disease progression. **: $p<0.01$

POC volumetric analysis

We firstly manual marked the region of interest (ROI) of the POC on a T1-weighted whole-brain ICBM2009c template (www.bic.mni.mcgill.ca/ServicesAtlases/ICBM152NLin2009). The POC was defined based on Dr. John Price's work on primates. Specifically, the POC included cortical areas that receive direct axons from the olfactory bulb. The regions included the anterior olfactory nucleus (antOlf), piriform cortex, olfactory tubercle, anterior most portions of the periamygdaloid and entorhinal cortex, and medial amygdaloid nucleus. We then projected the POC ROI in the standard space onto individual subject space using the FSL software (https://fsl.fmrib.ox.ac.uk/fsl/fslwiki) before calculating the individual POC volume.

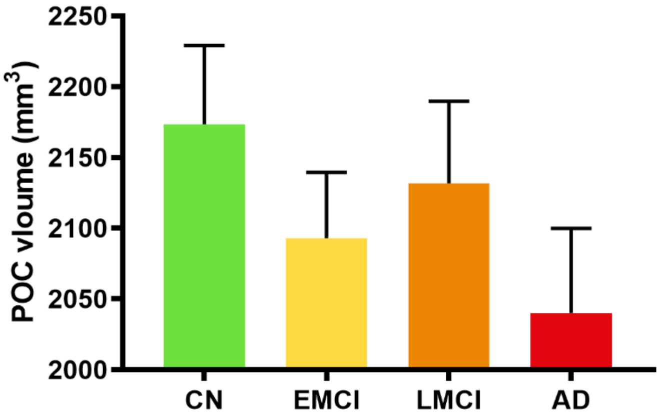

Figure S4. POC volume in CN (cognitively normal), EMCI (early mild cognitive impairment), LMCI (late mild cognitive impairment), and AD (Alzheimer's disease) groups. A decreasing trend is shown dependent on AD disease state.

1. Buckner, R.L., et al., Cortical hubs revealed by intrinsic functional connectivity: mapping, assessment of stability, and relation to Alzheimer's disease. Journal of neuroscience, 2009. 29(6): p. 1860-1873.
